# Impact of wearing external breast prosthesis on body posture of patients after unilateral mastectomy: a randomized controlled trial

**DOI:** 10.3389/fonc.2025.1456562

**Published:** 2025-03-11

**Authors:** HongMei Xie, XiaoQian Lan, YueHua Wang, QiuZhou Wang, Zi Ye, Hong Chen, Lan Fu

**Affiliations:** ^1^ Department of General Surgery, West China Hospital, Sichuan University, Chengdu, Sichuan, China; ^2^ Breast Disease Center, West China Hospital, Sichuan University, Chengdu, Sichuan, China; ^3^ West China School of Nursing, Sichuan University, Chengdu, Sichuan, China; ^4^ Department of Anesthesiology, West China Hospital, Sichuan University, Chengdu, Sichuan, China; ^5^ Department of Nursing, West China Hospital, Chengdu, Sichuan, China

**Keywords:** external breast prosthesis, mastectomy, unilateral breast cancer, body posture, shoulder asymmetry, scapular tilt

## Abstract

**Introduction:**

Unilateral mastectomy induces postural alterations; however, the resolution of this issue in clinical settings remains unknown. This study aimed to explore the effects of wearing external breast prosthesis on the posture of patients after unilateral mastectomy.

**Methods:**

A total of 240 patients who underwent unilateral mastectomy for breast cancer in our hospital’s breast surgery department from September 2020 to March 2021 were selected, and they were registered and randomized in a 1:1 ratio to receive one of two treatments: (1) the intervention group wearing a external breast prosthesis (similar in weight to the breast) and (2) the control group wearing a cotton breast prosthesis (almost no weight). The generalized estimating equation method was used to analyze the impact of wearing external breast prosthesis on the patients’ body posture 3 and 6 months after the intervention.

**Results:**

Statistically significant differences were observed between the two groups regarding forward head posture, shoulder asymmetry, scapular tilt, and neck tilt (P < 0.05). However, the two groups had no significant differences in scapular adduction/abduction, pelvic tilt, and trunk inclination (P > 0.05). Over time, all degrees of deviation in postural abnormalities exhibited an upward trend, with postural abnormalities becoming increasingly serious.

**Discussion:**

External breast prosthesis can improve postural abnormalities in patients with forward head posture, shoulder asymmetry, and scapular and neck tilts. However, there was no significant improvement in the short-term body posture of the patients concerning scapular adduction/abduction, pelvic tilt, or trunk inclination, indicating that further research is required to understand the effects of wearing external breast prosthesis on patients’ body posture.

**China clinical trial registry:**

https://www.chictr.org.cn/showproj.html?proj=56939, identifier ChiCTR2000040897.

## 1 Introduction

According to the latest data from the International Agency for Research on Cancer (1), the global incidence rate of breast cancer in 2020 (11.7%) has surpassed that of lung cancer (11.4%), making breast cancer the most common cancer among women. In the same year, 420,000 newly diagnosed breast cancer cases were reported in China, accounting for 9.1% of all new cancer cases, making it one of the most prevalent cancers among Chinese women. With the advancement of medical technology and the widespread availability of cancer screening, the mortality and 5-year survival of breast cancer in Chinese women are 4% and 83.2%, respectively (1, 2). Unilateral breast cancer is more common, and 70% of patients with breast cancer prefer mastectomy as the primary surgical method. However, due to limitations in medical technology, individual disease conditions, and traditional psychological beliefs, the rate of breast reconstruction is only 9.6% (3). Although mastectomy can achieve the therapeutic effect of tumor removal, it can also affect the breast appearance, especially after unilateral mastectomy. The asymmetry between the left and right sides of the body and the imbalance in chest wall weight not only affects a woman’s external appearance, causing body image concerns, but also disrupt the gravitational balance of the body, leading to postural instability and resulting in serious physiological, psychological, and social harm to patients during the recovery period (4, 5).

Women’s breasts have a significant impact on body posture and balance (6, 7), and the absence of breasts after unilateral mastectomy can lead to severe bodily functional issues and postural abnormalities (8–12). After unilateral mastectomy, 82.3% of patients exhibit incorrect body posture compared to healthy women (7). The loss of breast weight on one side affects the even distribution of body weight, causing changes in the gravimetry and biomechanics of posture on both sides of the body. The body’s center of gravity and gravity line tilt toward the non-operative side, affecting the symmetry of the shoulders, scapulae, and neck, leading to forward head posture, shoulder asymmetry, scapular tilt, and neck tilt, resulting in functional posture disorders postoperatively (7, 13–15).

Suitable prosthesis weight can provide symmetry, maintain body balance, and improve postural abnormalities. However, whether the weight of the prosthesis affects early postoperative body posture remains controversial (16–18). Currently, 59%-67% of patients in China wear traditional cotton breast prostheses and do not choose heavier external breast prosthesis because they are expensive (19–21). Research has shown that 63.3% of patients wear external breast prosthesis only in social situations (22), while 15.7% of patients wear external breast prosthesis continuously. Patients in a previous study wore external breast prosthesis for a short period and lightweight cotton breast prostheses for longer (20). Further investigation of the influence of breast prosthesis weight on body posture is required. Therefore, we conducted a randomized controlled trial to explore the effect of the prosthesis weight on patients’ body posture after unilateral mastectomy.

## 2 Materials and methods

### 2.1 Design and setting

This study was a randomized controlled trial. Patients with breast cancer who underwent wound suture removal in the breast surgery dressing room of our hospital were recruited between September 2020 and March 2021. The research protocol was sanctioned by the Institutional Review Board of the West China Hospital of Sichuan University under approval number 2019 (564) and was subsequently registered with the China Clinical Trial Registry under registration number ChiCTR2000040897. Written informed consent was obtained from all participants prior to their inclusion in the study.

### 2.2 Participants

Breast cancer patients who underwent mastectomy on the breast surgery ward were recruited. The inclusion criteria were as follows: (1) between 18 and 60 years of age; (2) conscious and able to communicate verbally or in writing; (3) diagnosed with unilateral breast cancer by imaging and pathological histology; (4) unilateral mastectomy with or without lymph node dissection; (5) no visible abnormal spine morphology or postural abnormalities. The exclusion criteria were: (1) diagnosis of neurological, skeletal, or rheumatic disorders, or other diseases severely affecting posture; and (2) history of bodily injuries, such as spinal, shoulder, and neck injuries. In addition, the following additional exclusion criteria were applied during the course of the study: (1) non-operative factors causing postural changes during the study, such as fractures and bodily injuries, and (2) failure to complete follow-up for any reason.

### 2.3 Sample size

Eligible patients were registered and randomized in a 1:1 ratio to receive one of two treatments: (1) the intervention group using a external breast prosthesis (similar in weight to the breast) and (2) the control group using a cotton breast prosthesis (almost no weight). The primary endpoint was the degree of deviation in shoulder asymmetry. According to the literature review and pre-experimental results, the mean degree of deviation of shoulder asymmetry in the control group is 5.69 ± 4.10 mm, and it is expected that the degree of deviation of shoulder asymmetry in the intervention group can be reduced by 1.92 mm. A sample size of 96 patients per group was estimated to provide at least 90% power and a 2-sided type I error rate of 5% to detect the degree of deviation in shoulder asymmetry. The sample size was increased by 20% to account f-or dropouts and ineligible patients. A sample size of 120 patients per group was required, resulting in a total sample size of 240.

### 2.4 Research procedure

**Patient approach:** After unilateral mastectomy, patients with breast cancer usually stay at our hospital for 6–7 days. During the dressing change on the day of discharge, the nurse responsible for the dressing change provided general information about the study to the patients. Those interested in participating in the study were instructed to contact the nurse in charge of enrollment and received detailed information about the survey in person. All patients provided written informed consent. Eligible patients were registered and randomized in a 1:1 ratio into the intervention and control groups.

**Postural assessment general requirements:** Postural assessment was performed in a warm, separate, concealed room. The patients were asked to tie up their hair, expose their earlobes, and remove upper-body clothing. Additionally, during the postural assessment, patients were instructed to stand with their arms hanging naturally by their sides, their heads held naturally upright, their chins slightly extended, and their backs against a body-posture-assessment wall chart, while looking straight ahead.

**Instruments for postural assesment:** A posture assessment wall chart, a soft ruler, a triangular ruler, writing pens, markers, an electronic scale, and a sociometer were used as assessment instruments.

**Postural assessment methods:** Seven postural parameters were measured: two in the sagittal plane (forward head posture and trunk rotation angle) and five in the coronal plane (neck tilt, shoulder asymmetry, scapular asymmetry, scapular asymmetry relative to the spine, and pelvic tilt). This study’s measurements were conducted collaboratively by nurses and rehabilitation therapists. The aforementioned postural parameters were measured by a trained nurse using the human body posture assessment method described in Johnson’s *Postural Assessment*, with close supervision by a rehabilitation physician (23). To reduce the risk of bias in the measurement process, the measurements were conducted by two nurses with over five years of extensive clinical experience. Rehabilitation therapists conducted uniform supervision, training, and evaluation for the nurses responsible for measurements. We conducted a Kappa coefficient test, with a Kappa coefficient of 0.80. First, the patients’ body weight and height were measured to calculate their body mass index (BMI). Next, anatomical points easily located on the skin surface were marked while the patient was standing, including the acromion, earlobe, inferior angles of the scapula, spinous process, and iliac crest. The postural assessment methods are detailed in **Table 1**.

**Table 1:** Postural assessment methods (23).

Postural abnormalities	Method	Picture
Forward head posture	The back and heels were placed closely against the body posture assessment wall chart in the standing position. The distances from the earlobe to the wall (d_1_) and from the acromion to the wall (d_2_) were measured. The relative distance of the forward head posture was calculated using the following formula (mm): *d*_relative distance_ =½*d*_1_-*d*_2_½	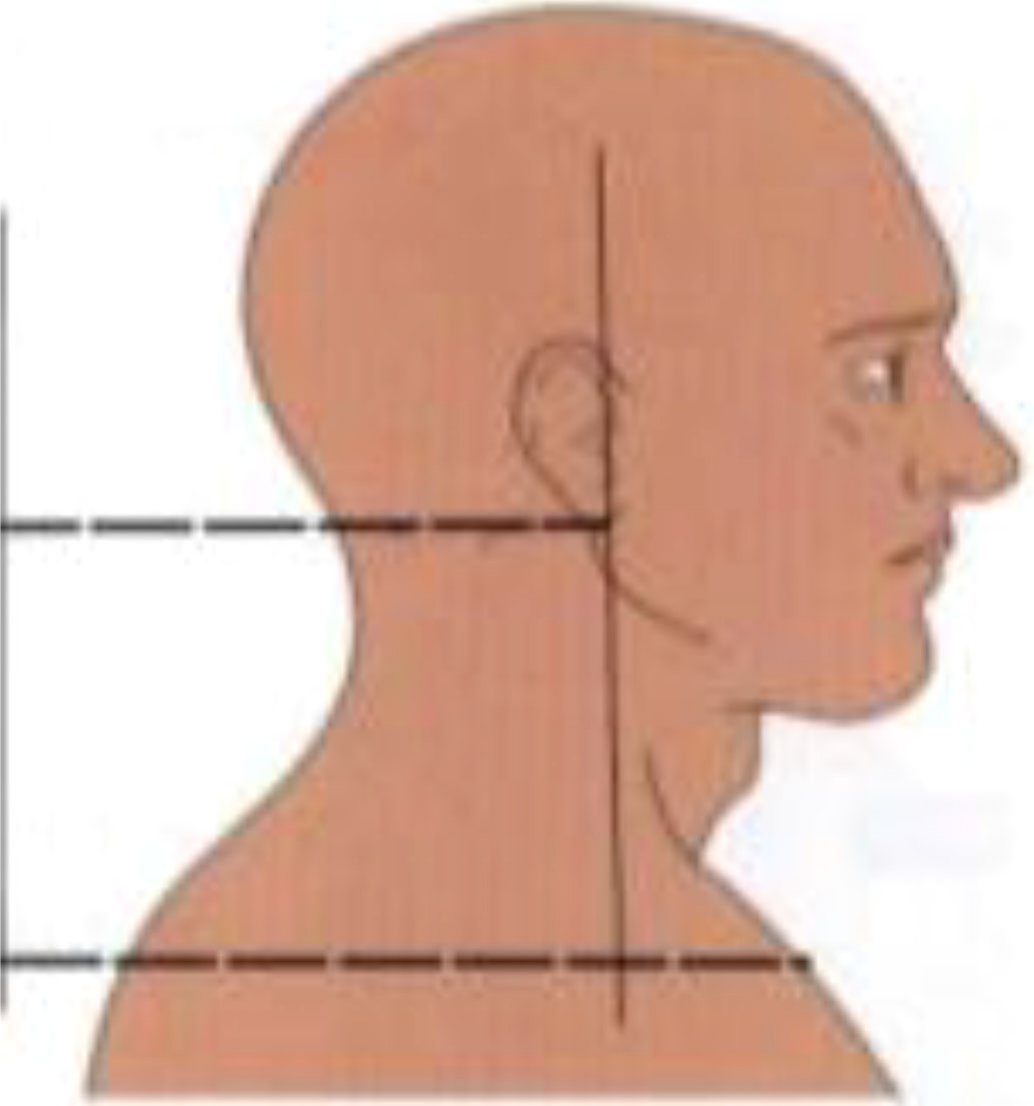
Neck tilt	The distances from the earlobe to the acromion were measured on each side using a soft ruler, and the relative distance was calculated using the following formula (mm): *d _relative distance_* =|*d*_mastectomy s_ *_ide_ - _d non-mastectomy side_* |	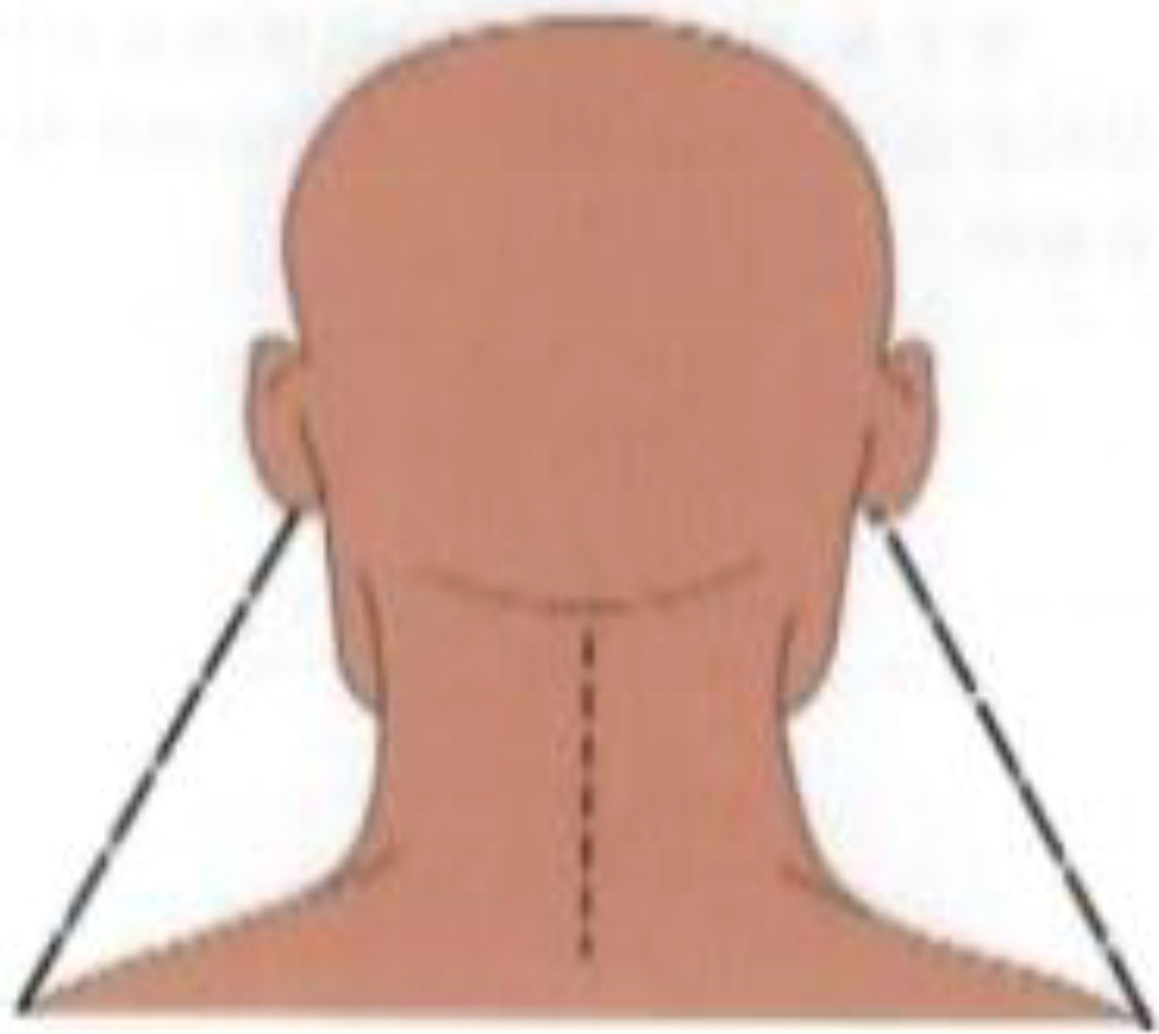
Shoulder asymmetry	A triangular ruler was used to measure the relative height of the acromion (mm).	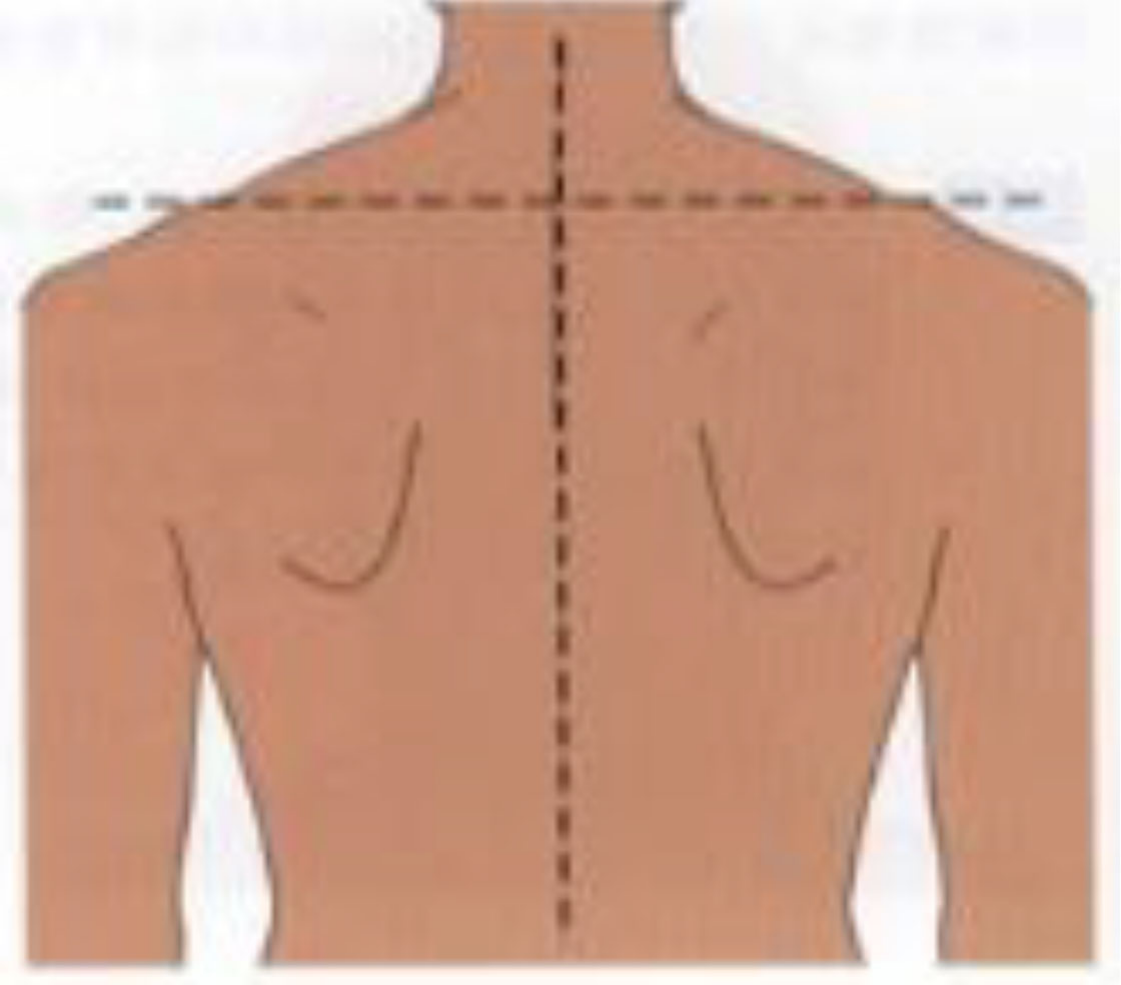
Scapula asymmetry	A triangular ruler was used to measure the relative height of the lower subscapular angle (mm).	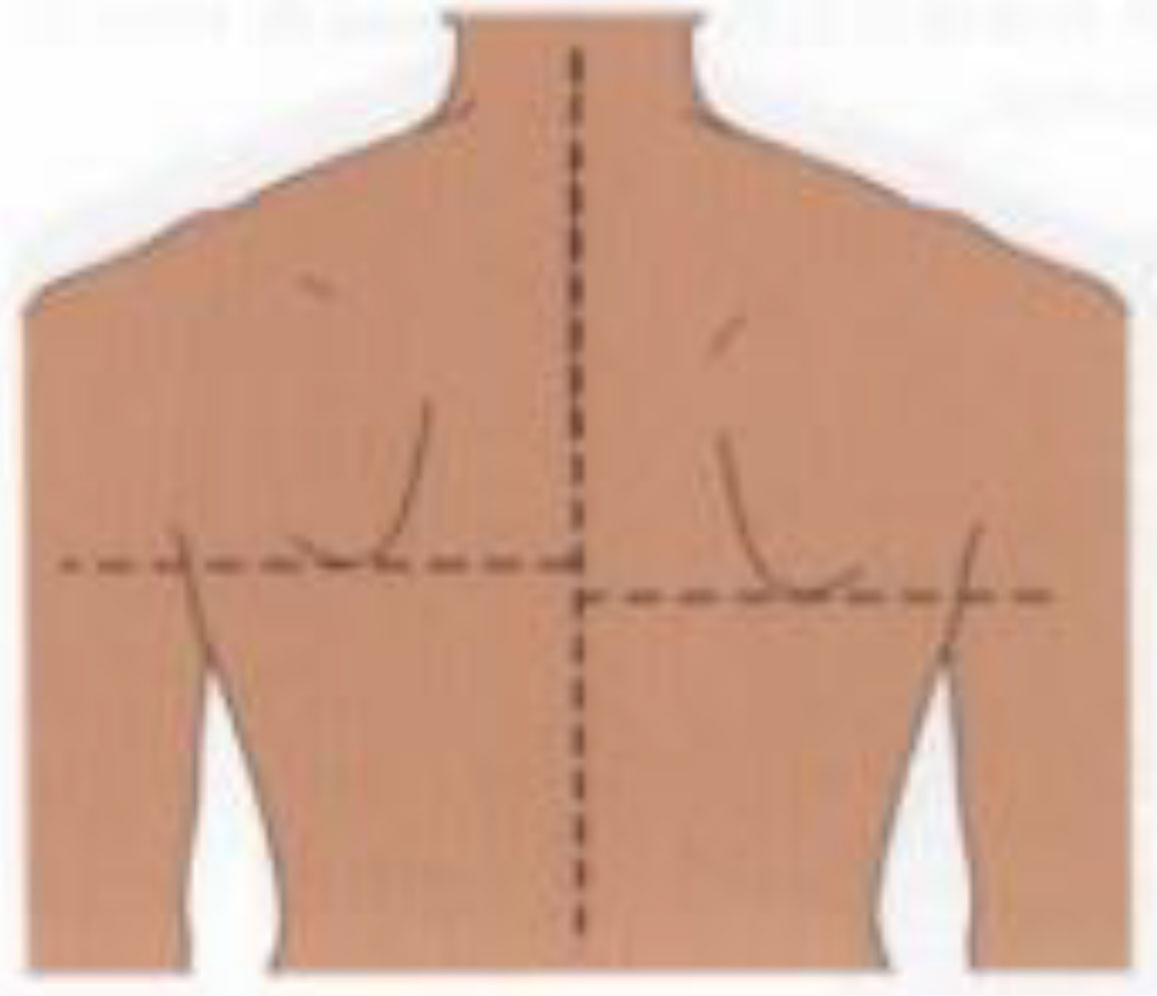
Deviation of scapular adduction/abduction (scapula asymmetry relative to the spine)	A triangular ruler was used to measure the relative distance of the lower subscapular angle to the spine (mm).	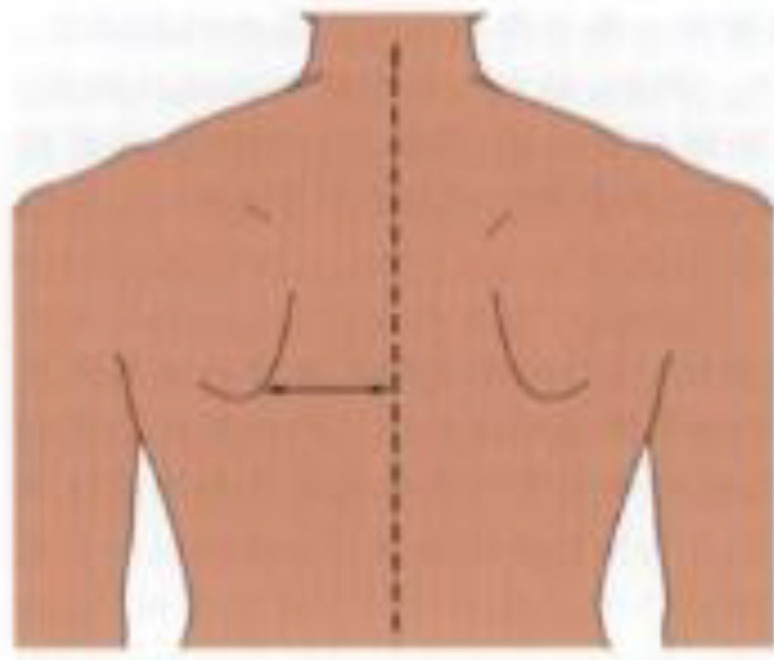
Pelvic tilt	A triangular ruler was used to measure the relative height of the iliac crests (mm).	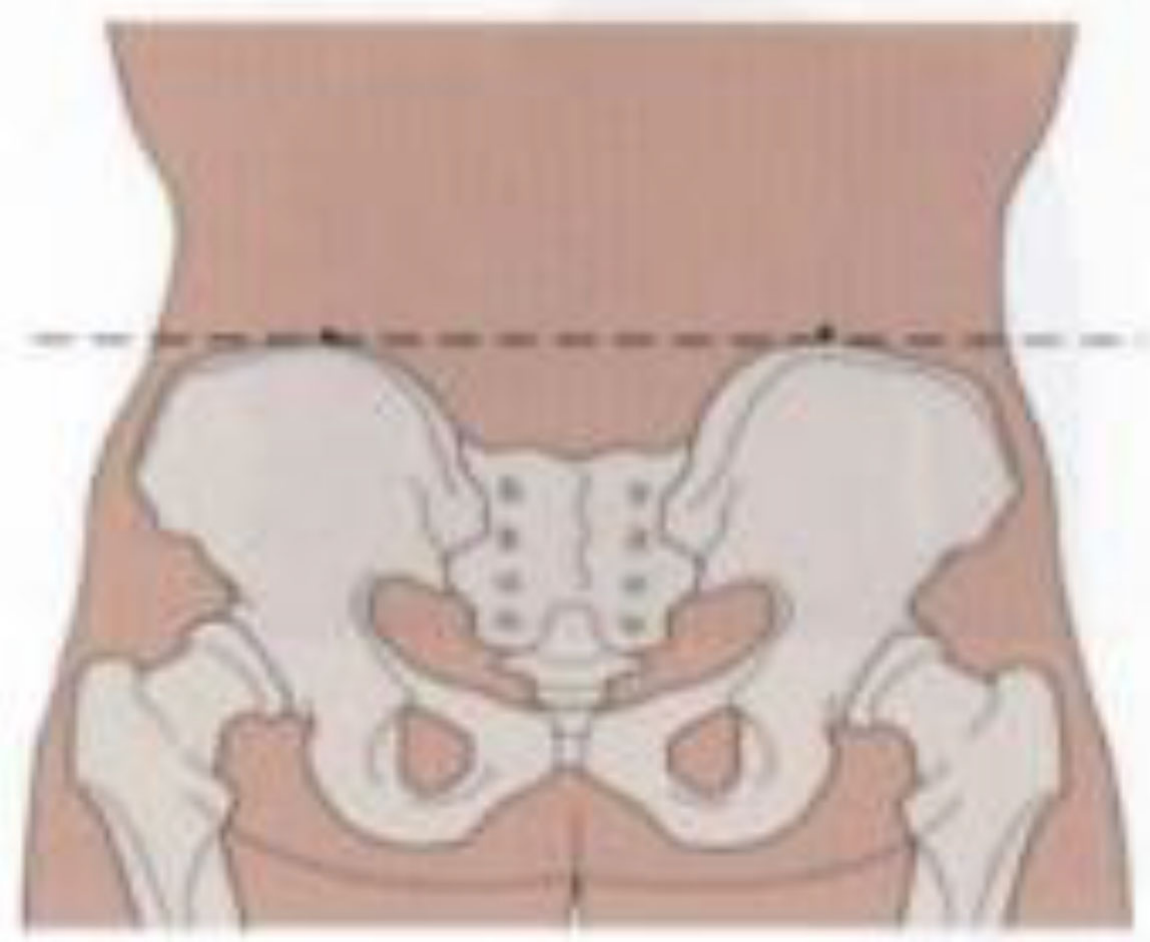
Trunk rotation angle	Adam’s forward bending test (FBT) was performed with the feet placed together, knees straight, while bending at the hips to nearly 90° with the arms freely hanging forward and palms joined together. The trunk rotation angle (°) was measured in this position using a scoliometer. Adam’s FBT position.	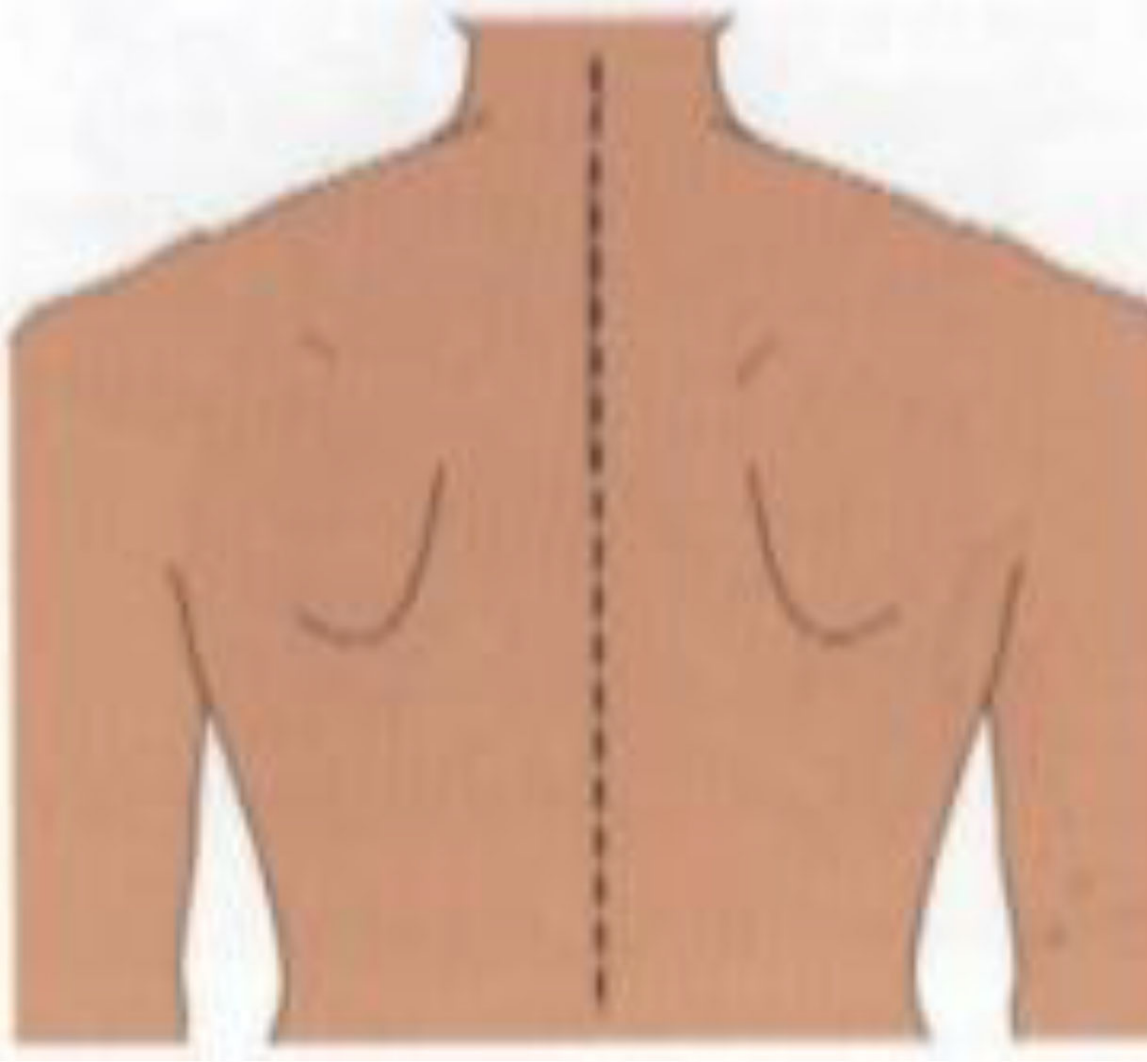

All pictures were taken from *Postural Assessment* written by Jane Johnson (23). This was approved by the author and publisher.

**Data collection and follow-up:** Demographic and clinical details of the patients were retrieved from electronic medical records. Postural assessments were conducted at three time points: baseline (the day of suture removal), and 3 and 6 months after the intervention. If the patient was unable to return to the breast surgery ward at the required time, she was allowed to complete the postural assessment one week before or one week later.

### 2.5 Minimization of protocol breaches

We implemented various measures to minimize protocol breaches, as the study required patients to return to the hospital regularly for posture assessments. These measures included: (1) encouraging family members to provide support to the patients throughout the study; (2) recruiting patients who resided as close to the hospital as possible to minimize commuting difficulties; (3) setting up a specific group in WeChat, a popular Chinese social media platform, to address any potential barriers and enhance adherence to the protocol; (4) regularly contacting each patient via phone to assess their recovery after unilateral mastectomy and provide appropriate advice; and (5) assisting patients in scheduling appointments with the surgeon as needed.

### 2.6 Statistical analysis

Data analysis was conducted using SPSS 26.0 software (IBM Corp., Armonk, NY, USA). Descriptive statistics, including frequencies and proportions, were employed to characterize the study sample. Generalized estimating equations were utilized to assess the impact of wearing breast prostheses of varying weights on body posture following unilateral mastectomy, with time as the primary factor and postural parameters as repeated measures. All tests were two-tailed, and the α level was set at P<0.05.

## 3 Results

### 3.1 Flow of participants through the trial and baseline characteristics

**Figure 1:**
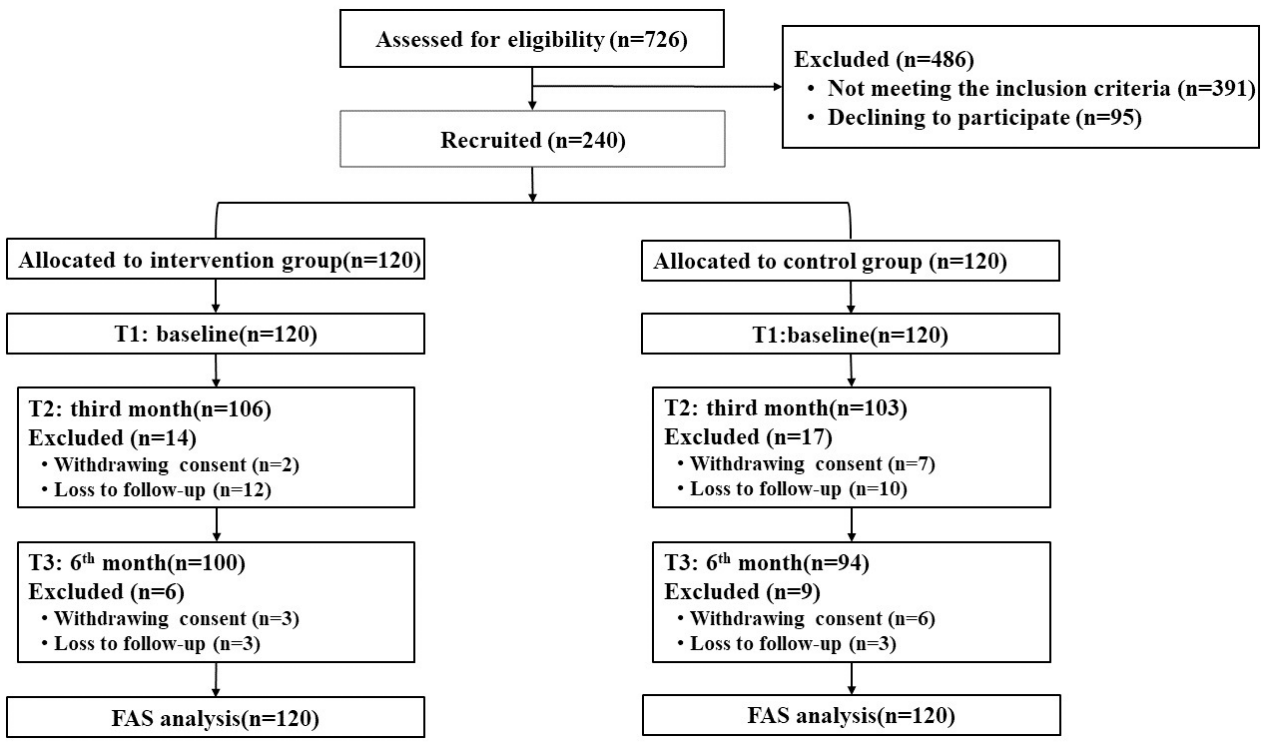
Study flow diagram.

The study was conducted from September 2020 to March 2021. Out of the 726 patients initially recruited, 486 were excluded. The remaining 240 patients with complete data were included in the final analysis (**Figure 1**). The two groups showed no statistical differences in terms of general characteristics (P>0.05). **Table 2** presents the baseline characteristics of the patients included in the study.

**Table 2:** Baseline characteristics (N=240).

Characteristics	Experimental (n=120)	Control (n=120)	z/χ^2^/t	*P*
Age (Years); M (P25, P75)	48 (44, 55)	52 (45, 56)	-1.78^a^	0.075
Education level (n, %)				
Primary school/below	8 (6.70)	8 (6.70)	1.19^a^	0.233
Junior high school	36 (30.00)	46 (38.30)		
Senior high school	37 (30.80)	31 (25.83)		
College or above	39 (32.50)	35 (29.17)		
Marital status (n, %)				
Married	104 (86.70)	106 (88.40)	1.01^b^	0.796
Divorced	12 (10.00)	12 (10.00)		
Widowed/Never married	4 (3.30)	2 (1.60)		
Employment status (n, %)				
Employed	30 (25.00)	20 (16.70)	4.83^b^	0.184
Medical leave due to illness	20 (16.70)	14 (11.60)		
Retired	33 (27.50)	38 (31.70)		
Unemployed	37 (30.80)	48 (40.00)		
Family monthly income (n, %)				
<1000 CNY	7 (5.83)	16 (13.33)	1.02^a^	0.303
1000-3000 CNY	42 (35.00)	35 (29.17)		
3001-5000 CNY	29 (24.17)	34 (28.33)		
5001-8000 CNY	23 (19.17)	19 (15.84)		
>8000 CNY	19 (15.83)	16 (13.33)		
Body mass index (kg/m^2^, *Mean ± SD*)	24.17 ± 3.27	23.95 ± 2.76	0.57^c^	0.565
chest circumference (cm); M (P_25_, P_75_)	91.00 (84.25, 98.00)	90.00 (84.00, 96.00)	1.24^a^	0.211
Waist circumference (cm); M (P_25_, P_75_)	80.00 (76.00, 87.00)	80.00 (74.00, 83.75)	1.51^a^	0.130
Mastectomy weight (g); M (P_25_, P_75_)	454.70 (261.27, 675.00)	409.00 (243.25, 670.25)	0.52^a^	0.599
Operative side (n, %)				
Right	62 (51.67)	62 (51.67)	0.00^b^	1.000
Left	58 (48.33)	58 (48.33)		
Dominant hand (n, %)				
Right	118 (98.33)	119 (99.17)	0.33^b^	0.561
Left	2 (1.67)	1 (0.83)		

CNY, Chinese Yuan; ^a^z value; ^b^χ2 value; ^c^t value.

### 3.2 Measurement indicators of body posture parameters

#### 3.2.1 Degree of deviation of head forward posture

**Figure 2:**
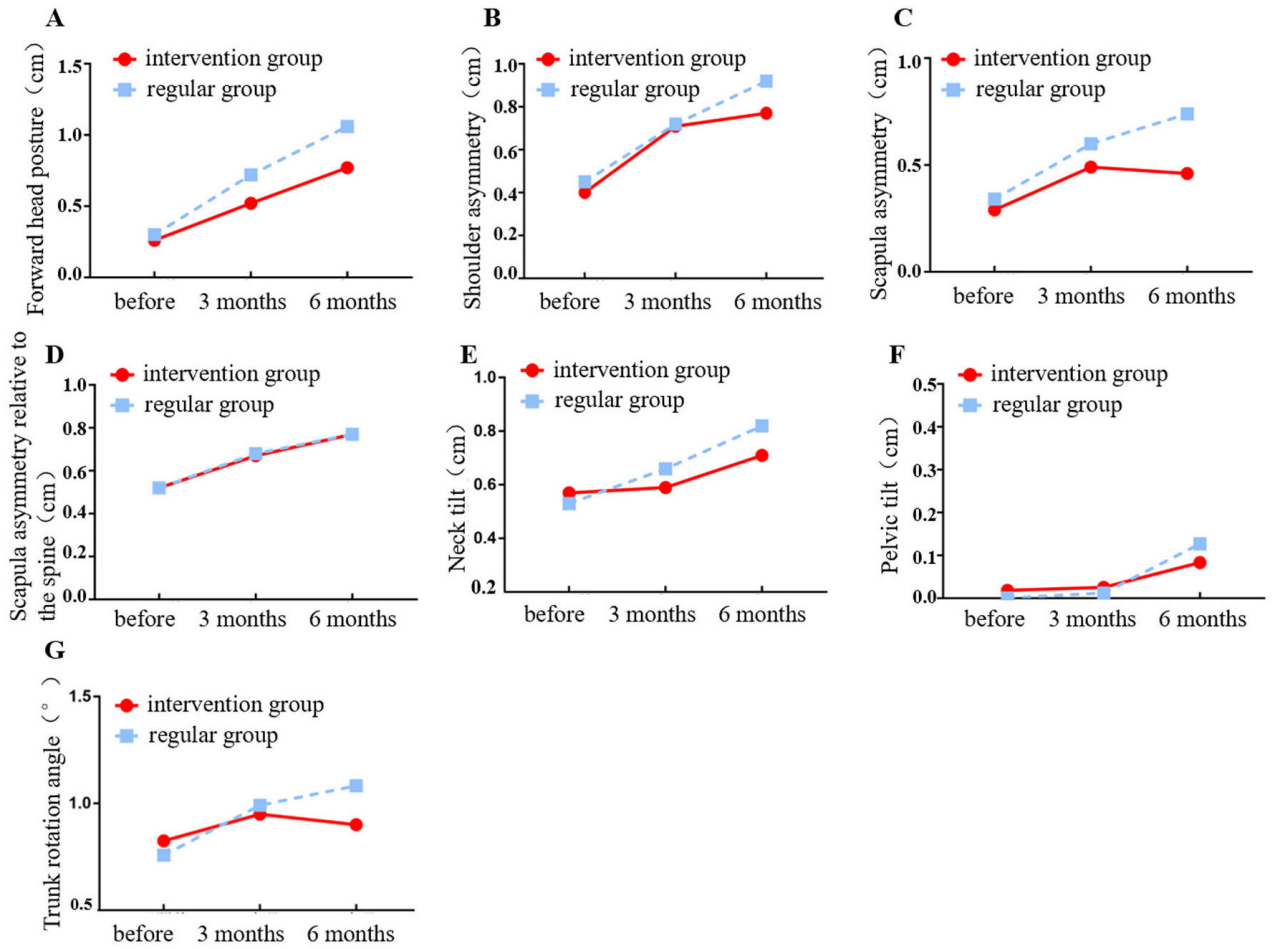
Changing trajectory of the degree of deviation of postural abnormalities over time. **(A)** Forward head posture; **(B)** Shoulder asymmetry; **(C)** Scapula asymmetry; **(D)** Scapula asymmetry relative to the spine; **(E)** Neck tilt; **(F)** Pelvic tilt; **(G)** Trunk rotation angle.

The degree of deviation in forward head posture in both patient groups changed over time, with statistically significant differences (P<0.001). There was a statistically significant difference after the intervention by treatment factors (P<0.001) and an interaction effect between grouping and time factors (P<0.05). Three and six months after the intervention, the degree of deviation in forward head posture between the two groups showed statistically significant differences (P<0.001). Detailed data are shown in **Table 3** and **Figure 2A**.

**Table 3:** Degree of deviation of postural abnormalities (N=240, cm, M, Waldχ^2^) .

Variable	Group	Baseline	3th month	6th month	Time effect	Intergroup effect	Interaction effect
Forward head posture	Experimental(n=120)	0.2 (0, 0.5)	0.5 (0.3, 0.8)	0.8 (0.42, 1)	339.955^**^	11.331^**^	10.135^*^
	Control(n=120)	0.4 (0, 0.5)	0.6 (0.5, 1)	1 (0.6, 1.5)			
	*Z*	-1.888	-3.259	-3.753			
	*P*	0.058	0.001*	<0.001^**^			
Shoulder asymmetry	Experimental(n=120)	0.5 (0, 0.5)	0.5 (0.3, 1)	0.5 (0.5, 1)	142.647^**^	2.363	11.877^*^
	Control(n=120)	0.5 (0.2, 0.5)	0.6 (0.5, 1)	1 (0.5, 1)			
	*Z*	-1.047	-0.795	-2.803			
	*P*	0.294	0.426	0.005^*^			
Scapula asymmetry	Experimental(n=120)	0.2 (0, 0.5)	0.5 (0, 0.8)	0.5 (0, 1)	81.351^**^	12.864^**^	15.063^**^
	Control(n=120)	0.5 (0, 0.5)	0.5 (0.5, 1)	1 (0.5, 1)			
	*Z*	-1.165	-2.507	4.8			
	*P*	0.243	0.012^*^	0.000^**^			
Scapula asymmetry relative to the spine	Experimental(n=120)	0.5 (0, 1)	0.5 (0.22, 1)	0.6 (0.5, 1)	43.451^**^	0.001	0.030
	Control(n=120)	0.5 (0, 1)	0.55 (0.20, 1)	0.8 (0.5, 1)			
	*Z*	-0.076	-0.322	-0.021			
	*P*	0.938	0.746	0.982			
Neck tilt	Experimental(n=120)	0.5 (0, 1)	0.5 (0, 1)	0.5 (0.3, 1)	30.802**	0.798	6.915^*^
	Control(n=120)	0.5 (0, 1)	0.5 (0.2, 1)	0.8 (0.5, 1)			
	*Z*	-0.154	-1.127	-1.969			
	*P*	0.877	0.259	0.048^*^			
Pelvic tilt	Experimental(n=120)	0 (0, 0)	0 (0, 0)	0 (0, 0)	28.167^**^	0.082	2.966
	Control(n=120)	0 (0, 0)	0 (0, 0)	0 (0, 0)			
	*Z*	-1.739	-0.462	-1.595			
	*P*	0.081	0.643	0.110			
Trunk rotation angle	Experimental(n=120)	1 (0, 1)	1 (0, 1)	1 (0, 1)	15.840^**^	0.367	4.417
	Control(n=120)	1 (0, 1)	1 (0, 2)	1 (0, 2)			
	*Z*	-1.175	-0.343	-1.447			
	*P*	0.239	0.731	0.147			

^*^ *P*<0.05; ^**^ *P*<0.001.

#### 3.2.2 Degree of deviation of shoulder asymmetry

The degree of deviation in shoulder asymmetry in both patient groups varied over time with statistically significant changes (P<0.001). In the post-treatment intervention factor, there were no significant statistical differences (P>0.05), but there were interaction effects between the group and time factors (P<0.05). Three months after the intervention, the degree of deviation in shoulder asymmetry between the two groups was not statistically significant (P>0.05). However, six months after the intervention, the difference was statistically significant (P<0.05). Detailed data are shown in **Table 3** and **Figure 2B**.

#### 3.2.3 Degree of deviation of scapular asymmetry

The degree of deviation in scapular asymmetry between both patient groups changed over time with statistically significant differences (P<0.001). There was a statistically significant difference after the treatment factor intervention (P<0.001) and an interaction effect between the grouping and time factors (P<0.001). Three and six months after the intervention, the degree of deviation in scapular asymmetry between the two groups showed statistically significant differences (P<0.05). Detailed data are shown in **Table 3** and **Figure 2C**.

#### 3.2.4 Degree of deviation of scapular adduction/abduction

The degree of deviation in scapular adduction/abduction between the two groups varied over time, showing statistically significant changes (P<0.001). Post-treatment factor intervention, there was no statistically significant difference (P>0.05), nor was there any interaction effect between the group and time factors (P>0.05). Three and six months after the intervention, there were no statistically significant differences in the degree of scapular adduction/abduction deviation between the two groups (P>0.05). Detailed data are shown in **Table 3** and **Figure 2D**.

#### 3.2.5 Degree of deviation of neck tilt

The degree of deviation in neck tilt between the two groups of patients changed over time, showing statistically significant variations (P<0.001). Post-treatment factor intervention, there was no statistically significant difference (P>0.05), but there was interaction effect between the group and time factors (P<0.05). Three months after the intervention, there were no statistically significant differences in the degree of scapu-lar adduction/abduction deviation between the two groups (P>0.05). However, at 6 months post-intervention, the comparison showed statistica-lly significant differences (P<0.05). Detailed data are shown in **Table 3** and **Figure 2E**.

#### 3.2.6 Degree of deviation of pelvic tilt

The degree of deviation in the pelvic tilt between the two groups of patients altered over time, showing statistically significant variations (P<0.001). The post-treatment factor of intervention did not show significant statistical differences (P>0.05), and there was no interaction effect between the group and time factors (P>0.05). Three and six months after the intervention, there were no statistically significant differences in the degree of pelvic tilt deviation between the two groups (P>0.05). Detailed data are shown in **Table 3** and **Figure 2F**.

#### 3.2.7 Degree of deviation of trunk rotation angle

The degree of deviation in the trunk rotation angle between the two groups of patients altered over time, showing statistically significant variations (P<0.001). The post-treatment factor of intervention did not show significant statistical differences (P>0.05), and there was no interaction effect between the group and time factors (P>0.05). Three and six months after the intervention, there were no statistically significant differences in the degree of deviation of the trunk rotation angle between the two groups (P>0.05). The detailed data are shown in **Table 3** and **Figure 2G**.

## 4 Discussion

### 4.1 Improvement to the degree of deviation of forward head posture

In this study, the degree of deviation in forward head posture was generally small in both groups but showed an upward trend over time. The degree of deviation in forward head posture in the intervention group was lower than that in the control group. The differences in the degree of deviation of forward head posture between the two groups at 3 and 6 months post-intervention were statistically significant (P<0.05), consistent with studies by Jetha (16) and Manikowska (24). The theory of gravitational imbalance states that when a person stands naturally, the body’s center of gravity is slightly in front of the sacrum, and the head, neck, and trunk are on the same gravitational line (25). External breast prosthesis are similar in weight to the missing breast of the patient and can maintain the body’s gravitational line and center of gravity unchanged, thereby achieving sagittal plane balance. In contrast, cotton breast prostheses are lighter than external breast prosthesis and cannot be used to correct uneven patient weight distribution. The body adopts a series of sequential twisting movement patterns to maintain balance and stability, such as the forward extension of the head, neck, and shoulders, to maintain sagittal plane balance (26), eventually resulting in a functional forward head posture. Without timely intervention, this can lead to structural forward bending of the cervical spine, resulting in an irreversible abnormal posture. This suggests that researchers should guide patients to choose appropriate external breast prosthesis and wear them as early as possible to prevent forward displacement of the gravitational line caused by uneven weight distribution and to improve the forward head’s abnormal posture.

### 4.2 Improvement to the degree of deviation of shoulder asymmetry

The degree of deviation of shoulder asymmetry was generally low in both groups but showed an upward trend over time. The degree of shoulder asymmetry deviation was lower in the intervention group than in the control group. There was no statistically significant difference in the degree of deviation in shoulder asymmetry between the two groups at 3 months after the intervention (P>0.05). In contrast, there was a statistically significant difference in the degree of deviation in shoulder asymmetry between the two groups 6 months after the intervention (P<0.05). This indicates that external breast prosthesis can improve the level of shoulder peaks in patients and prevent the occurrence of shoulder asymmetry 6 months after the intervention, which is consistent with the results of studies by Ciesla (15) and Hojan (27). Ciesla et al. (15) found that the deviation in shoulder asymmetry in the external breast prosthesis group was significantly lower than that in the unilateral mastectomy group in the 6th month after surgery. At 18 months postoperatively, the difference in the degree of deviation of shoulder asymmetry between the two groups was greatest. At 24 months postoperatively, owing to the body’s compensatory mechanisms, the changes in both groups were small, similar to the results of this study. This suggests that further longitudinal studies should be conducted to explore the intervention effects of external breast prosthesis on the degree of deviation in shoulder asymmetry at different periods to provide a reference for selecting appropriate breast prostheses in the future.

### 4.3 Improvement to the degree of deviation of scapular tilt

The degree of deviation of scapular tilt was generally low in both groups but showed an upward trend over time. The degree of scapular tilt deviation in the intervention group was lower than that in the control group, and the differences in the degree of scapular tilt deviation between the two groups at 3 and 6 months post-intervention were statistically significant (P<0.05). This indicates that the early wearing of external breast prosthesis can effectively prevent the occurrence of scapular tilt, which is consistent with the results of the Crosbie study (28). Scapular kinematics and the theory of gravitational imbalance suggest that after unilateral mastectomy, an imbalance in the weight of one breast causes the scapula on the affected side to rotate upward and the body’s gravitational line to shift to the healthy side (28). The relative height of the scapula can be affected when the flexibility of one side of the glenohumeral joint is limited. The weight of external breast prosthesis can help maintain the humeral head within the glenoid fossa as much as possible, improve the flexibility of the glenohumeral joint, reduce the relative height of the inferior angles of the scapulae on both sides of the body, and prevent scapular tilt (7, 29).

### 4.4 Improvement to the degree of deviation of neck tilt

The degree of neck tilt deviation in the intervention group was lower than that in the control group, and the differences in the neck tilt between the two groups at 6 months post-intervention were statistically significant (P<0.05). This suggests that the early wearing of external breast prosthesis after unilateral mastectomy can effectively improve the degree of deviation in neck tilt. Nicoletti’s (30) study found that a 400 g external breast prosthesis is the optimal load threshold for maintaining physiological balance of cervical spine posture, similar to the weight of the external breast prosthesis used in this study. Therefore, external breast prosthesis are effective in maintaining the physiological balance of the cervical spine. However, in the third month of intervention, there was no statistically significant difference in the degree of neck tilt deviation between the two groups (P>0.05). This suggests that the effect of the external breast prosthesis requires a considerable amount of time to become evident. Researchers can advise patients to increase their wear time or extend the follow-up period to enhance the intervention effects.

### 4.5 Improvement to the degree of deviation of scapular adduction/abduction, pelvic tilt, and trunk rotation angle

The degree of deviation in scapular adduction/abduction, pelvic tilt, and trunk rotation angle was generally low in both groups, and these parameters showed an upward trend in both groups over time. However, there were no statistically significant differences in the degree of deviation of scapular adduction/abduction, pelvic tilt, or trunk rotation angle between the intervention and control groups (P>0.05). This indicates that the early use of external breast prosthesis does not improve the degree of deviation of the scapular adduction/abduction, pelvic tilt, or trunk rotation angle, which is inconsistent with the results of a study by Koralewska (31). This may be because the follow-up period in this study was only six months, which was relatively short. At the same time, although weight loss in one breast resulted in muscle imbalance and tension in the shoulder and chest muscles, the impact on the weight-bearing mechanism of the spine and pelvis in patients was relatively small. Koralewska et al. found that the degree of forward tilt deviation and trunk lordosis in the external breast prosthesis group was lower than that in the unilateral mastectomy group (31). With prolonged follow-up, the improvement effect of the external breast prosthesis became more pronounced. However, in the 6th month after the intervention, the two groups had no significant difference in the degree of deviation of scapular adduction/abduction, pelvic tilt, and trunk rotation angle. This may be because the breast weight of Asian patients is lower than that of other patients, and the weight of the external breast prosthesis has a smaller impact on the main weight-bearing mechanisms of the human body, such as the spine or pelvis. Scapular adduction/abduction and trunk rotation reflect the position of the spinal gravity line. An imbalance of the spinal gravity line requires long-term muscle imbalance to cause structural changes in the spine and skeleton, forming irreversible abnormalities in body posture.

## 5 Conclusions

In conclusion, early use of external breast prosthesis can improve abnormal body posture of forward head posture, high-low shoulder, scapular tilt, and neck tilt after unilateral mastectomy. However, there is no significant improvement in the short-term abnormal body postures of scapular adduction/abduction, pelvic tilt, or trunk rotation angle. This study suggests that researchers may extend the follow-up time for interventions and increase the sample size to further investigate the long-term effects of external breast prosthesis on the body posture of patients after unilateral mastectomy.

## Data Availability

The original contributions presented in the study are included in the article/supplementary material. Further inquiries can be directed to the corresponding authors.
